# Inflammatory Pathway Genes Belong to Major Targets of Persistent Organic Pollutants in Adipose Cells

**DOI:** 10.1289/ehp.1104282

**Published:** 2012-01-19

**Authors:** Min Ji Kim, Véronique Pelloux, Erwan Guyot, Joan Tordjman, Linh-Chi Bui, Aline Chevallier, Claude Forest, Chantal Benelli, Karine Clément, Robert Barouki

**Affiliations:** 1 INSERM UMR-S 747, Université Paris Descartes, Centre Universitaire des Saints-Pères, Paris, France; 2 INSERM U872, Nutriomique team 7, Université Pierre et Marie Curie-Paris 6, Centre de Recherche des Cordeliers, UMR S 872, Paris, France; 3 Assistance Publique-Hôpitaux de Paris, Institute of Cardiometabolism and Nutrition, Pitié-Salpêtrière, Paris, France; 4 Assistance Publique-Hôpitaux de Paris, Hôpital Necker-Enfants Malades, Paris, France

**Keywords:** adipocytes, adipose tissue, inflammation, obesity, PCB, persistent organic pollutants, TCDD

## Abstract

Background: Epidemiological studies emphasize the possible role of persistent organic pollutants (POPs) in obesity and the metabolic syndrome. These pollutants are stored in adipose tissue (AT).

Objectives: Our aim was to study the effects of POPs on human adipose cells and rodent AT.

Methods: Using human multipotent adipose-derived stem cells, we carried out large-scale gene expression analysis to identify the major pathways modified by 2,3,7,8-tetrachlorodibenzo-*p*-dioxin (TCDD), polychlorinated biphenyl (PCB) congener 126 (PCB-126), and PCB-153 and to evaluate their toxic effects. The effects of TCDD on gene expression and AT histology were also assessed in mice.

Results: The most significantly regulated genes in both precursor cells and adipocytes were those involved in the inflammatory/immune response, cancer, and metabolism pathways. Interestingly, the fold induction and the number of modulated genes were higher in precursors than in adipocytes, suggesting that the former could be more sensitive to the effect of pollutants. When cells were treated with combinations of pollutants, the effects of the AhR ligands TCDD and PCB-126 were dominant compared with those of the non-dioxin-like PCB-153. The effects of AhR ligands were reduced by the AhR antagonist α-naphthoflavone. The regulation of inflammatory pathway was observed in wild-type AT but not in *AhR*-knockout mice.

Conclusions: Both *in vitro* and *in vivo* studies showed that adipose cells were targets of AhR ligands and suggest that inflammation is one of the main regulated pathways. These observations suggest a possible contribution of pollutants to low-grade AT inflammation that accompanies the pathogenesis of metabolic diseases.

Several recent epidemiological studies have emphasized the possible role of environmental pollutants in obesity and the metabolic syndrome. The serum concentrations of persistent organic pollutants (POPs) such as polychlorinated dibenzo-*p*-dioxins and dibenzofurans (PCDD/Fs), polychlorinated biphenyls (PCBs), and organochlorine pesticides are reported to be associated with body mass index, elevated triglycerides, glucose intolerance, and cardiovascular diseases (Airaksinen et al. 2011; Alonso-Magdalena et al. 2011; Ha et al. 2007; Uemura et al. 2009). Molecular and cellular mechanisms of 2,3,7,8-tetrachlorodibenzo-*p*-dioxin (TCDD), the most potent compound of PCDD/Fs, are well characterized. TCDD was shown to induce xenobiotic-metabolizing enzymes as well as other genes involved in cell proliferation, differentiation, apoptosis, and inflammation (Haarmann-Stemmann et al. 2009). Most TCDD effects are mediated by its binding to the aryl hydrocarbon receptor (AhR), leading to AhR nuclear translocation, heterodimerization with aryl hydrocarbon receptor nuclear translocator (ARNT), and binding to xenobiotic responsive elements present in the promoters of target genes (Barouki et al. 2007; Puga et al. 2009). The mechanisms of action of other dioxins, furans, and certain PCBs such as PCB-126 are thought to be similar to those of TCDD, albeit with different potencies (Denison et al. 2002). In contrast, the mechanisms of action of other PCBs such as PCB-153 are poorly understood.

Adipose tissue (AT) is particularly relevant for the assessment of POP effects. POPs are lipophilic compounds stored primarily in the fat mass, which accounts for their long half-life (approximately 10 years). Pharmacokinetic studies suggest that this is the case for TCDD, particularly at low environmentally relevant doses (Emond et al. 2006). Furthermore, kinetic studies indicate that fat mass is among the major determinants of POP apparent half-lives in humans (Milbrath et al. 2009). Although this storage function may be protective to a certain extent, it also leads to a chronic low-grade internal exposure of humans to these toxicants (Kim et al. 2010). Furthermore, AT has important endocrine and metabolic physiological functions and could itself be a privileged target of these pollutants. Metabolic diseases and obesity are accompanied by a major alteration of AT functions such as a state of chronic low-grade inflammation in which adipocytes and other AT cells (e.g., endothelial cells, immune cells, preadipocytes) play a role by producing inflammation-related mediators (Goldberg 2009). These inflammatory molecules contribute to marked perturbation of paracrine dialogues between those cells, which contributes to altered AT biology, promoting, for example, local inflammation and metabolic alteration such as insulin resistance as well as increased remodeling and fibrosis (Dalmas et al. 2011). Stienstra et al. (2011) have suggested that the resulting deregulation in the secretion of some inflammatory mediators in the systemic circulation could contribute to numerous obesity comorbidities, such as cancers. Hence, it is critical to assess the effect of POPs on AT. Some studies reported antiadipogenic and proinflammatory effects of TCDD and the dioxin-like PCB-77 on mouse 3T3-L1 cells (a mouse embryonic fibroblast cell line) (Arsenescu et al. 2008; Wen et al. 2007) or mouse embryo fibroblasts (Alexander et al. 1998). TCDD was also found to impair insulin action, signaling, and secretion in insulin-sensitive cells such as beta cells and adipocytes (Hsu et al. 2010; Nishiumi et al. 2010; Piaggi et al. 2007). Limited studies have been devoted to human adipocytes.

Our aim was to evaluate the effects of AhR ligands on adipocyte functions. We first identified human multipotent adipose-derived stem (hMADS) cells as an appropriate model to study the effect of pollutants on human adipocytes (Rodriguez et al. 2005a, 2005b; Zaragosi et al. 2006). We carried out large-scale gene expression analyses to target the major pathways and genes that were altered by pollutants. We focused our studies on TCDD, PCB-126 (the most potent of the dioxin-like PCBs), and PCB-153 (the most abundant of the non-dioxin-like PCBs). We studied the effects of these POPs on both precursor cells and mature adipocytes. We also characterized the effect of dioxin on AT in wild-type (WT) and *AhR*-knockout (KO) mice.

## Materials and Methods

*Cell culture.* Cell culture media were purchased from Invitrogen (Cergy-Pontoise, France), human fibroblast growth factor 2 (hFGF2) from Peprotech (London, UK), and all the other products from Sigma-Aldrich (Saint Quentin Fallavier, France).

The establishment and characterization of hMADS cells have been described previously (Rodriguez et al. 2005a). Proliferating cells were seeded in Dulbecco’s modified Eagle’s medium (DMEM) with 10% fetal calf serum, 2.5 ng/mL hFGF2, 10 mM HEPES buffer, 50 U/mL penicillin, and 50 μg/mL streptomycin and kept at 37°C in 5% CO_2_. hFGF2 was removed when cells reached confluence. The next day, cells were incubated in differentiation medium: DMEM/Ham’s F-12 medium containing 10 μg/mL transferrin, 5 μg/mL insulin, 0.2 nM triiodothyronine, 100 μM 3-isobutyl-1-methylxanthine (IBMX), 1 μM dexamethasone (DEX), and 0.5 μg/mL rosiglitazone. At day 3, DEX and IBMX were omitted from the differential medium until day 10. Differentiation (i.e., neutral lipid accumulation) was assessed by Oil Red O staining as described elsewhere (Rodriguez et al. 2005a). The experiments were carried out between days 11 and 13 while the cells were treated for 48 hr with 25 nM TCDD, 1 μM PCB-126, or 10 μM PCB-153, all dissolved in dimethyl sulfoxide (DMSO). For the 48-hr treatment of precursor cells, the proliferating cells were treated at confluence by the same compounds, at the same concentrations.

*Animals.* Homozygotic WT mice (C57BL/6) were purchased from Charles River Laboratories (Arbresle, France), and the inactivation of the *AhR* gene was performed to produce *AhR*-KO mice as described elsewhere (Fernandez-Salguero et al. 1995). Mice were maintained on a standard 12-hr light/dark cycle with *ad libitum* access to standard chow and water. Seven- to 8-week-old male mice were treated with a single intraperitoneal injection of 10 μg/kg TCDD dissolved in corn oil (Sigma-Aldrich) or vehicle control (corn oil). At 48 hr or 72 hr after intraperitoneal injection, the mice were killed by asphyxiation with CO_2_. This dose of TCDD was selected because previous studies in rodents suggested that it led to AT concentrations that were within the range of those used in our *in vitro* studies (Wang et al. 2000). The animals were treated humanely and with regard for alleviation of suffering.

*Determination of mRNA Levels.* Total RNA was extracted from undifferentiated or *in vitro* differentiated adipocytes with the RNeasy Total RNA Mini Kit (Qiagen, Courtaboeuf, France) and from mouse AT with RNeasy Lipid Tissue Mini Kit (Qiagen). Total RNA concentration was assessed spectrophotometrically with a NanoDrop spectrophotometer (Thermo Scientific, Wilmington, DE, USA), and their quality was determined with the Agilent 2100 Bioanalyzer (Agilent Technologies, Massy, France). Total RNA (1 μg) was reverse transcribed using a high-capacity cDNA reverse transcription kit (Applied Biosystems, Courtaboeuf, France). Real-time polymerase chain reactions (PCRs) were conducted with ABsolute™ QPCR SYBR® Green ROX mix (Thermo Electron SAS, Courtaboeuf, France) on an Applied Biosystems 7900HT Fast Real-Time PCR System (Applied Biosystems). mRNA values were normalized to the expression level of a reference gene. We tested hypoxanthine-guanine-phosphoribosyltransferase (HPRT), TATA box binding protein (TBP), and ribosomal protein L13A. HPRT had the most stable expression level among different conditions in precursor cells and mouse AT, and TBP had the most stable expression level in adipocytes. Primer sequences are available upon request.

*Immunohistochemistry.* Epididymal AT biopsies were fixed overnight at 4°C in 4% paraformaldehyde and then processed for standard paraffin embedding. Sections of 5 μm were stained as described below and observed under a Zeiss 20 Axiostar Plus microscope (Zeiss, Oberkochen, Germany). Digital images were captured by tri-CCD camera (DXC-390P; Sony, Paris, France).

*Adipocyte diameter.* Slides were stained with hematoxylin and eosin. Adipocyte diameters were measured using PerfectImage software, version 7.4 (Claravision, Massy, France). We estimated the diameter of all adjacent adipocytes from each of four separate photographs of the same slide in parenchyma. These measurements were performed at 10× magnification, with 200–300 adipocyte diameters measured for each biopsy to calculate the mean and SE for each sample.

*Macrophages accumulation.* Anti-mouse F4/80 antibody (AbD Serotec, Düsseldorf, Germany) was used to detect macrophages (Khazen et al. 2005). Immunohistochemical detection was performed with the avidin–biotin peroxidase method as described previously (Cancello et al. 2006). Slides were counterstained with Mayer’s hematoxylin. Adipocytes and F4/80-positive cells were counted in 10 different randomly chosen areas in each processed slide at 40× magnification. The number of F4/80 cells per 100 adipocytes was considered the number of infiltrating AT macrophages.

*Cytokine measurements.* Cytokine concentrations in supernatants of cultured undifferentiated cells and adipocytes were determined by Luminex assay with a custom kit, Human Adipocyte Multiplex Immunoassay Kit–FOUR PLEX (Millipore, Saint Quentin-en-Yvelines, France) according to the manufacturer’s protocols using the Luminex xMAP Multiplexing Technology platform (Hôpital Saint Antoine, Paris, France).

*Sample preparation for microarray analyses.* Total RNA (200 ng) from each sample (each condition in quadruplicate) was amplified and transcribed into fluorescent cRNA with the Low RNA Input Linear Amplification Kit (Agilent Technologies). Cyanine-5 dye was incorporated into the control (DMSO treated) sample, whereas samples treated with TCDD, PCB-126, or PCB-153 were labeled with cyanine-3 dye. Samples were then hybridized to Agilent whole human genome microarrays (Whole Human Genome Microarray Kit, 4x44K). Sample preparation, hybridization, and microarray washing were performed according to the manufacturer’s recommendations. Arrays were scanned with a GenePix 4000B scanner (Axon Instruments-Molecular Devices, Sunnyvale, CA, USA).

*Statistical analysis.* For all microarrays, after loess normalization of log-transformed microarray data with the use of the Goulphar package (version 1.1.3; http://transcriptome.ens.fr/goulphar/index.php) (Lemoine et al. 2006), gene lists were filtered to identify genes common to all microarrays (i.e., 100% retrieval). Differential gene expression, using a 5% false discovery rate, was assessed with the use of the Significance Analysis of Microarrays (SAM; version 3.02) procedure (http://www-stat.stanford.edu/~tibs/SAM/). The functional profiling of gene expression data was determined using FunNet (http://www.funnet.info), which was described elsewhere (Prifti et al. 2008). The complete data set is publicly available at the National Center for Biotechnology Information Gene Expression Omnibus (http://www.ncbi.nlm.nih.gov/geo/) through series accession number GSE32026 and is also available on request from the authors.

For quantitative reverse transcriptase (qRT) PCR gene expression and cytokine measurements, Kruskal–Wallis and/or Mann–Whitney *U*-test using StatView 5.0 (SAS Institute Inc., Cary, NC, USA) was performed. Results are presented as the mean ± SE. *p*-Values < 0.05 were considered significant.

## Results

*hMADS, a valuable human adipocyte model to evaluate pollutant toxicity.* hMADS exhibit features of stem cells, that is, a high ability to self-renew and the capacity to differentiate into different lineages, including adipocytes [see Supplemental Material, Figure 1 (http://dx.doi.org/10.1289/ehp.1104282)]. The differentiated cells exhibit the unique characteristics of human adipocytes, unlike rodent preadipocyte cell lines, for example, hMADS can secrete leptin and adiponectin. Furthermore, cytochrome P450 1B1 (CYP1B1), a constitutively expressed CYP1 family member in extrahepatic tissues, is potently induced by TCDD in both precursor and differentiated cells [see Supplemental Material, Tables 1, 2 (http://dx.doi.org/10.1289/ehp.1104282)]. AhR is also expressed in these cells. Thus, this cell line appears to be an adequate model for the assessment of the toxic effect of pollutants on human undifferentiated and differentiated adipocytes.

**Figure 1:**
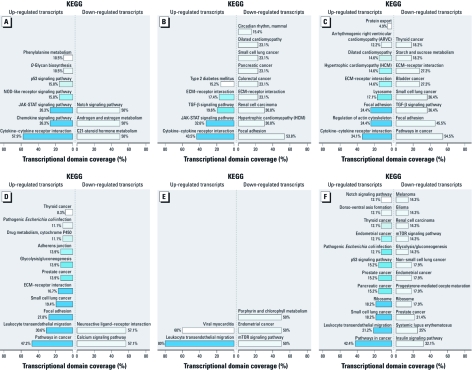
Functional profiling of microarray data determined by FunNet. (*A*–*C*) Precursor hMADS cells treated with 25 nM TCDD (*A*), 1 μM PCB-126 (*B*), or 10 μM PCB-153 (*C*) for 48 hr. (*D*–*F*) Differentiated hMADS cells treated with 25 nM TCDD (*D*), 1 μM PCB-126 (*E*), or 10 μM PCB-153 (*F*) for 48 hr. Abbreviations: ECM, extracellular matrix; mTOR, mammalian target of rapamycin; NOD, nucleotide-binding oligomerization domain; TGFβ, transforming growth factor-β.

**Table 1:** Role of AhR in pro- and anti-inflammatory gene regulation in hMADS precursors and adipocytes: fold induction after treatment (mean ± SE).

Gene	DMSO	α-NF	TCDD	TCDD + α-NF	PCB-126	PCB-126 + α-NF
Precursor cells												
*CYP1B1*		1.01 ± 0.10		2.01 ± 0.21		9.29 ± 1.42		3.70 ± 0.65*		9.67 ± 1.61		4.61 ± 0.97*
*IL1B*		1.04 ± 0.18		1.84 ± 0.24		11.09 ± 1.11		3.04 ± 0.36*		8.93 ± 1.15		3.14 ± 0.27*
*IL8*		1.04 ± 0.21		1.64 ± 0.33		4.10 ± 0.82		1.93 ± 0.38*		4.18 ± 1.29		2.09 ± 0.13*
*MCP1*		1.01 ± 0.09		1.16 ± 0.14		2.62 ± 0.13		1.32 ± 0.21*		2.53 ± 0.12		1.33 ± 0.24*
*PAI2*		1.03 ± 0.17		2.01 ± 0.39		21.18 ± 4.40		2.92 ± 0.45*		14.98 ± 3.77		4.29 ± 0.78*
*PTGS2*		1.00 ± 0.03		1.43 ± 0.07		6.75 ± 1.29		2.29 ± 0.10*		5.78 ± 0.24		2.69 ± 0.31*
*IL1ra*		0.97 ± 0.29		1.54 ± 0.18		2.58 ± 0.66		1.01 ± 0.04*		2.21 ± 0.25		1.26 ± 0.13*
Adipocytes												
*CYP1B1*		1.03 ± 0.17		0.72 ± 0.09		7.16 ± 1.67		1.89 ± 0.11*		6.94 ± 1.46		2.70 ± 0.60*
*IL1B*		1.04 ± 0.20		0.93 ± 0.07		1.00 ± 0.19		1.21 ± 0.18		1.31 ± 0.27		1.28 ± 0.16
*IL8*		1.02 ± 0.14		0.81 ± 0.16		1.81 ± 0.20		0.93 ± 0.11*		2.26 ± 0.96		1.25 ± 0.29
*MCP1*		1.07 ± 0.29		0.92 ± 0.07		1.88 ± 0.26		1.12 ± 0.16*		1.57 ± 0.32		1.16 ± 0.16
*PAI2*		1.01 ± 0.09		0.55 ± 0.16		3.93 ± 0.44		1.64 ± 0.33*		4.83 ± 0.52		2.30 ± 0.66*
*PTGS2*		1.03 ± 0.19		0.84 ± 0.10		1.63 ± 0.38		1.00 ± 0.12		2.07 ± 0.30		1.14 ± 0.02*
*IL1ra*		1.00 ± 0.05		1.09 ± 0.25		0.96 ± 0.00		1.23 ± 0.12		1.14 ± 0.01		1.35 ± 0.31
Gene expression was normalized by HPRT expression in undifferentiated cells and by TBP expression in adipocytes. *p < 0.05, For TCDD-treated or PCB-126–treated cells without versus with pretreatment with α-NF, by Mann–Whitney *U*-test.												

*Large-scale studies and major gene regulation by TCDD, PCB-126, or PCB-153.* Preliminary studies allowed us to determine the amount of pollutants to be used in *in vitro* experiments. We chose the amount that resulted in a statistically significant induction of selected target genes without inducing cellular toxicity [measured by the MTT [3-(4,5-dimethylthiazol-2-yl)-2,5-diphenyltetrazolium bromide] assay; data not shown]. Gene expression was monitored by large-scale microarray analysis comparing treated and untreated cells. The sets of genes differentially modulated by each pollutant were determined by SAM with a false discovery rate of 5%. In precursor cells, the expression of 236, 1,344, and 792 genes was up-regulated and that of 17, 158, and 104 genes was down-regulated by TCDD, PCB-126, and PCB-153, respectively. In adipocytes, the expression of 477, 117, and 441 genes was up-regulated and that of 153, 13, and 399 genes was down-regulated by TCDD, PCB-126, and PCB-153, respectively (data not shown, available upon request). To identify the enrichment in functional groups in up- and down-regulated gene lists, we used the FunNet tool. According to KEGG (Kyoto Encyclopedia of Genes and Genomes; http://www.genome.jp/kegg/) (Kanehisa and Goto 2000; Kanehisa et al. 2011) pathways, the inflammatory response was strongly induced by all the pollutants in precursor cells. Chemokine signaling pathway, cytokine–cytokine receptor interaction, and the JAK-STAT signaling pathway represented more than 50% of up-regulated transcripts in TCDD-treated and PCB-126–treated cells (Figure 1A,B). Other pathways, such as cell adhesion, metabolism, and cancer pathways, were also modulated by all pollutants (Figure 1A–C).

In adipocytes, results suggested that cancer-related pathways were modulated by pollutants (Figure 1D–F); in particular, those involved in leukocyte transendothelial migration pathway were up-regulated by all three compounds. Because leukocyte migration from blood to tissues is important for the inflammatory response, this is in agreement with the increased inflammation observed in AT after pollutant treatment.

*Determination of effects of pollutants by qRT-PCR.* We first validated the microarray observation by qRT-PCR by measuring the expression of genes involved in metabolism [*CYP1B1*, *CYP19A1*, fatty acid binding protein 4 (*FABP4*), NAD(P)H dehydrogenase, quinone 1 (*NQO1*)], cell adhesion or migration [intercellular adhesion molecule 1 (*ICAM1*), transforming growth factor, beta-induced (*TGFBI*), thrombospondin 1 (*THBS1*), vascular cell adhesion molecule 1 (*VCAM1*)], inflammation [chemokine (C-X-C motif) ligand 12 (*CXCL12*), interleukin 1β (*IL1B*), *IL8*, plasminogen activator inhibitor 2 (*PAI2*), signal transducer and activator of transcription 1 (*STAT1*), tumor necrosis factor receptor superfamily, member 11b (*TNFRSF11B*)], and cancer pathways [insulin-like growth factor binding protein 3 (*IGFBP3*), prostaglandin-endoperoxide synthase 2 (*PTGS2*), tribbles homolog 3 (*TRIB3*), wingless-type MMTV integration site family, member 5A (*WNT5A*)]. Both precursors and adipocytes were treated with TCDD, PCB-126, or PCB-153. Several mRNAs, such as those encoding *CYP1B1*, *IL8*, neuronal pentraxin 1 (*NPTX1*), *PAI2*, *PTGS2*, *NQO1*, *STAT1*, *TGFBI*, *THBS1*, *TNFRSF11B*, and *WNT5A*, were induced in both types of cells by the AhR ligands TCDD and PCB-126, and the fold induction of expression was generally higher in precursors than in adipocytes [see Supplemental Material, Tables 1 and 2 (http://dx.doi.org/10.1289/ehp.1104282)]. The level of transcripts encoding *CYP19A1*, *FABP4*, *ICAM1*, *IGFBP3*, *IL1B*, *TRIB3*, or *VCAM1* was modulated only in precursors. We also assessed the expression level of *AHR* mRNA because it was previously found to decrease during adipogenesis in 3T3-L1 cells, resulting in the loss of functional response to xenobiotics (Shimba et al. 1998). In hMADS, *AHR* expression was similar at the two cell stages and was significantly decreased by the treatment with AhR ligand in precursors (see Supplemental Material, Table 1). Thus, the greater sensitivity of precursor cells did not seem to be related to *AHR* expression level.

PCB-153 modulated the expression of some of the selected genes in precursors but not in adipocytes [see Supplemental Material, Tables 1 and 2 (http://dx.doi.org/10.1289/ehp.1104282)]. The effect of PCB-153 in precursors was similar to that of AhR ligands for *CYP19A1*, *ICAM1*, *PTGS2*, *TNFRSF11B*, and *VCAM1* and different for *CXCL12*, *IL1B*, *IGFBP3*, *NPTX1*, *STAT1*, *THBS1*, and *WNT5A*. In adipocytes, no regulation was observed.

**Figure 2:**
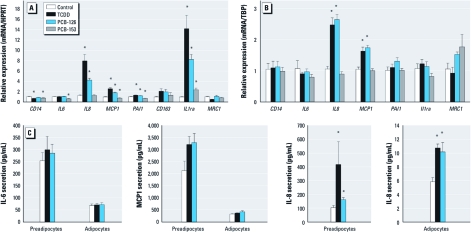
Regulation of cytokines involved in obesity-related inflammation. mRNA levels were assessed by qRT-PCR of commonly regulated pro- and anti-inflammatory cytokines in obesity-related low-grade inflammation in undifferentiated cells (*A*) and adipocytes (*B*) treated with a single concentration of TCDD (25 nM), PCB-126 (1 μM), or PCB-153 (10 μM). Gene expression was normalized by HPRT or TBP expression using the ΔΔCt method and is expressed as mean of fold induction ± SE. (*C*) Measurement of proinflammatory cytokine secretion by Luminex assay in the supernatants of undifferentiated cells or adipocytes treated with 25 nM TCDD or 1 μM PCB-126 for 48 hr. Data are expressed as mean ± SE. *p < 0.05, compared with control by Mann–Whitney *U*-test.

*Pro- and anti-inflammatory effects of TCDD, PCB-126, and PCB-153.* We further characterized the gene family of the inflammatory network by measuring the expression of additional pro- and anti-inflammatory genes involved in obesity-related low-grade inflammation in AT. We tested the regulation of the proinflammatory genes *CD14*, *IL6*, *IL8*, monocyte chemoattractant protein 1 (*MCP1*), *CCl2*, *PAI1*, and tumor necrosis factor-alpha (*TNF*α) and the anti-inflammatory genes *CD163*, interleukin 1 receptor antagonist (*IL1ra*), and *IL10*, all reported to code for secreted proteins that are up-regulated in obesity (Fain 2010). In precursors and adipocytes, *TNF*α and *IL10* mRNA were undetectable. *IL1ra* and *PAI1* mRNA were increased, whereas *CD14* mRNA was decreased, by TCDD and PCB-153 only in precursors (Figure 2A). *MCP1* and *IL8* mRNAs were increased by AhR ligands in both types of cells (Figure 2A,B). Thus, AhR ligands regulated the expression of genes encoding pro- and anti-inflammatory cytokines involved in the regulation of obesity-associated inflammation. In contrast, the pattern of gene regulation by PCB-153 was different from that of the other POPs: it elicited a decrease in the expression of *CD14*, *IL6*, *MCP1*, and *PAI1* genes and an increase in *IL1ra* only in precursor cells (Figure 2A). Because of its modest effects, we did not further pursue the characterization of PCB-153 effects.

To evaluate whether gene expression correlates with the corresponding proteins, we analyzed the secretion of IL1β, IL6, IL8, and MCP1 in supernatants of cultured precursors and adipocytes (Figure 2C). The secretion level of cytokines was higher in precursors than in adipocytes, and IL8 was significantly increased by a 48-hr treatment with TCDD or PCB-126. IL1β secretion was undetectable. IL6 and MCP1 secretion were not significantly changed by TCDD or PCB-126 treatment.

The involvement of AhR in the inflammatory response was evaluated with an antagonist of TCDD, α-naphthoflavone (α-NF). The induction of *CYP1B1*, a prototypical AhR target gene and of proinflammatory genes by TCDD or PCB-126 were significantly decreased when cells were pretreated with α-NF (Table 1), suggesting that these xenobiotics could regulate the inflammatory pathway in adipose cells via the AhR.

**Figure 3:**
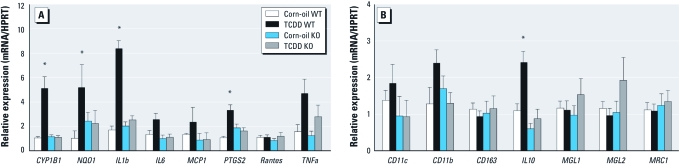
Modulation of inflammatory pathway genes in mouse AT. We evaluated the modulation of expression of proinflammatory (*A*) or anti-inflammatory (*B*) markers by qRT-PCR using the ΔΔCt method by reporting the differences between Ct of the target gene of TCDD-treated mice and the mean Ct of corn-oil–treated mice and by using *Hprt* gene expression as the reference in the epididymal AT of *AhR* WT or KO mice treated with 10 μg TCDD/kg or corn oil and sacrificed 48 hr later. Data are expressed as mean ± SE. For each gene, data were statistically compared by the Kruskal–Wallis test, and if significant differences were found, the Mann–Whitney *U*-test was applied. Rantes, Regulated upon Activation, Normal T-cell Expressed, and Secreted; also known as CCL5. *p < 0.05 compared with corn-oil–treated animals.

*AhR-dependent regulation of inflammatory genes in mouse AT.* Because of the importance of inflammation in the pathogenesis of obesity and metabolic disease, we studied the effect of AhR ligands *in vivo* on the epididymal AT of WT and *AhR*-KO mice. C57BL/6 male mice were treated with 10 μg/kg TCDD, a nonacutely toxic dose (Chapman and Schiller 1985) used in other similar studies (Schlezinger et al. 2010), and the expression of proinflammatory (Figure 3A) and anti-inflammatory (Figure 3B) markers was assessed. The expression of *Cyp1b1* and *Nqo1* genes that are typical AhR targets was induced in AT of TCDD-treated WT mice. Gene expression of the proinflammatory cytokines *Il1b* and *Ptgs2* and of the anti-inflammatory cytokine *Il10* was also increased in TCDD-treated animals. This modulation could not be observed in *AhR*-KO mice.

**Figure 4:**
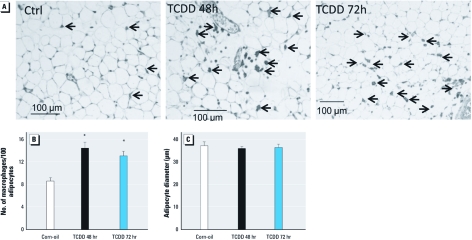
Immunohistochemistry of mouse AT. (*A* and *B*) The staining of infiltrated macrophages with F4/80 antibody was quantified by considering the number of macrophages (arrows) per 100 adipocytes. (*C*) Adipocyte diameter (μm) was determined by measuring 200–300 adipocytes in four different fields of the same slide. Data are mean + SE. Ctrl, control. *p < 0.05, Wilcoxon rank sum test between TCDD-treated and corn-oil–treated mice.

Because the local inflammation observed in the AT of obese subjects was generally correlated with the increased infiltration of macrophages and leukocytes (Dalmas et al. 2011), we examined mouse AT by immunohistology. Specific staining with F4/80 antibody showed an increased number of macrophages in TCDD-treated animals (Figure 4A,B). Because adipocyte hypertrophy is a factor determining macrophage infiltration in AT (Weisberg et al. 2003), we assessed the relationship between adipocyte diameter in TCDD-treated and corn-oil–treated mice. We found no difference in adipocyte size between these two conditions (Figure 4C).

These *in vivo* data are in agreement with the results obtained using hMADS cells. Indeed, TCDD was able to regulate the expression of genes encoding pro- and anti-inflammatory cytokines via AhR and to increase macrophage infiltration in AT.

## Discussion

Our results show that the hMADS cell line is a valuable model for the study of POP toxicity on human precursor and adipocytes because these cells respond to several pollutants at different differentiation stages. Observations made on these cells were confirmed *in vivo* by comparing AT from WT and *AhR*-KO mice. Some of our observations are in line with those made in human studies correlating POP levels with metabolic diseases (Airaksinen et al. 2011; Ha et al. 2007; Uemura et al. 2009). However, although the dioxin doses we used are common and nonacutely toxic, this exposure regimen is different from that of humans who are continuously exposed to very low levels of contaminants. Thus, although our functional data appear to have relevance to human observations, the difference in exposure should be kept in mind.

A nonsupervised analysis of the microarray data after treatment of precursors and adipocytes with POPs showed that transcripts for proteins involved in inflammation were among the most robustly regulated. To validate this results, we assessed the modulation of *ICAM1* (Brake et al. 2006), *NQO1* (Palming et al. 2007), *IGFBP3* (Chan et al. 2005), and *THBS1* (Varma et al. 2008) by POPs because, in addition to their role in cell adhesion, xenobiotic metabolism, angiogenesis, and cancer, these genes were reported to be related to adiposity, insulin resistance, and inflammation. We showed that AhR ligands were able to up-regulate the expression of these aforementioned genes. These data are in agreement with the *in vivo* metabolic disruption associated with dioxin-like POP and other POP levels (Airaksinen et al. 2011; Uemura et al. 2009) and may partly explain the growing evidence on the relationship between obesity and cancer (Harvey et al. 2011). Such associations could be also explained by insulin signaling impairment (Hsu et al. 2010; Nishiumi et al. 2010; Stienstra et al. 2011). However, the regulation of metabolic pathways appeared to be less prominent than that of the inflammatory pathway in our studies.

Obesity-associated inflammatory genes such as *IL1B*, *IL1ra*, *IL8*, and *PTGS2* (Goldberg 2009; Stienstra et al. 2011) were also increased by AhR ligands in hMADS cells. These results are in agreement with a recent study conducted on *in vitro* differentiated adipocytes from human bone marrow cells (Arsenescu et al. 2008; Li et al. 2008). We also showed that TCDD and PCB-126 gene regulation was mediated by AhR because it was potently inhibited by the AhR antagonist α-NF. In AT of TCDD-treated mice, we observed a similar up-regulation of transcripts for several inflammatory mediators (*Il1b*, *Il10*, *Ptgs2*) only in WT mice. Thus, our study suggests that the inflammatory pathway could be induced by TCDD via AhR. The modulation of gene expression was accompanied by an increased infiltration of macrophages and confirmed the existence of a TCDD-induced inflammatory state of AT. In animal models of obesity, a relationship was shown between adipose cell size and the concentration of macrophages in the stroma vascular fraction of AT (Weisberg et al. 2003). Such results were not found in the present study, suggesting that fat cell hypertrophy was not the sole determinant of macrophage recruitment into AT. Interestingly, a recent study found an association between small adipose cells and inflammation in moderately obese, weight-stable individuals (McLaughlin et al. 2010), suggesting that the association between adipocyte diameter and inflammation is more complex.

An important point revealed by our studies is that precursor cells appeared to be more sensitive than adipocytes to certain effects of AhR ligands. These data are in agreement with Fain (2010), who compared the release and expression of adipokines by nonfat versus fat cells and showed that the nonfat cells, containing adipose precursors, were more inflammatory than were fat cells. Several possible mechanisms could account for such observations. It is possible that adipocytes are less sensitive to AhR ligands because of their lipid compartment, which could divert these pollutants from their protein targets. Although we cannot exclude this possibility, it seems unlikely because the induction of *CYP1B1*, a prototypical AhR target gene, appears to be similar at both cell differentiation stages. Alternatively, there may be stage specificity of *AHR* transcriptional effects, highlighting the relevance of precursor cells in the mechanisms of pollutant-elicited AT toxicity.

PCB-153 is a non-AhR ligand that was shown to display some toxicity in other systems, notably as a cancer promoter (Knerr and Schrenk 2006), but the genomic biomarkers of PCB-153 exposure are not well identified. In hMADS, this pollutant displayed a moderate effect on differentiated cells. Interestingly, in undifferentiated cells, genes involved in cancer/inflammatory pathways such as *CXCL12*, *IGFBP3*, *IL1B*, and *WNT5A* were down-regulated by PCB-153, whereas they were up-regulated by AhR ligands. These observations highlight the critical role of AhR ligands in the modulation of inflammatory cytokines in adipocytes.

In conclusion, both our *in vitro* and *in vivo* studies suggest that adipocytes and their precursors are targets of POPs and that one of the main pathways that these pollutants trigger is inflammation. In view of the importance of this pathway in the pathogenicity of metabolic diseases, these observations indicate a possible mechanism of pollutant contribution to the development of these diseases.

## Supplemental Material

(283 KB) PDF
