# Supplementary material for: Inflammatory Pathway Genes Belong to Major Targets of Persistent Organic Pollutants in Adipose Cells
Source: Environ Health Perspect. 2012 Jan 19;120(4):508–14. doi: 10.1289/ehp.1104282 (PMC3339464; doi:10.1289/ehp.1104282)
Supplement: (283 KB) PDF [file ehp.1104282.s001.pdf]

Supplementary Material

Inflammatory Pathway Genes belong to Major Targets of Persistent Organic Pollutants in Adipose Cells

Min Ji Kim[1], Véronique Pelloux[2], Erwan Guyot[1], Joan Tordjman[2], Linh-Chi Bui[1], Aline Chevallier[1], Claude Forest[1], Chantal Benelli[1], Karine Clément[2], Robert Barouki[1]

[1] INSERM UMR-S 747, Paris, France; Université Paris Descartes, Centre Universitaire des Saints-Pères, Paris, France ; Assistance Publique-Hôpitaux de Paris, Hôpital Necker-Enfants Malades, 75015 France

[2] INSERM U872, Nutriomique team 7, Paris, 75006 France; Université Pierre et Marie Curie-Paris 6, Centre de Recherche des Cordeliers, UMR S 872, Paris, 75006 France; Assistance Publique-Hôpitaux de Paris, ICAN Institute of cardiometabolism and Nutrition, Pitié-Salpêtrière, Paris, 75013 France

Table of Contents

Supplementary Material, Table 1: Gene regulation by pollutants in undifferentiated hMADS cells... 3

Supplementary Material, Table 2: Gene regulation by pollutants in differentiated hMADS cells... 4

Supplementary Material, Figure 1: In vitro culture of hMADS cells 5

Supplementary Material, Table 1: Gene regulation by pollutants in undifferentiated hMADS cells

Gene	DMSO	TCDD	PCB126	PCB153
AhR	1.00 (0.05)	0.61 (0.01)*	0.63 (0.03)*	1.06 (0.05)
CYP1B1	0.99 (0.07)	7.29 (0.54)*	5.55 (0.40)*	0.94 (0.06)
CYP19A1	1.08 (0.13)	3.40 (0.39)*	2.22 (0.45)*	1.80 (0.14)*
NPTX1	1.06 (0.10)	24.56 (2.13)*	26.27 (2.17)*	0.69 (0.11)
NQO1	1.01 (0.08)	1.61 (0.16)*	1.33 (0.10)*	0.91 (0.05)
PAI2	1.03 (0.11)	24.41 (2.85)*	13.69 (1.37)*	1.26 (0.17)
FABP4	1.10 (0.20)	0.20 (0.04)*	0.40 (0.05)*	0.49 (0.12)*
IGFBP3	1.00 (0.03)	1.63 (0.09)*	1.70 (0.07)*	0.79 (0.07)*
STAT1	1.01 (0.05)	1.91 (0.197)*	1.69 (0.08)*	0.88 (0.03)
TGFβi	1.01 (0.05)	1.39 (0.05)*	1.26 (0.06)*	1.00 (0.05)
TSP1	1.01 (0.06)	1.39 (0.08)*	1.34 (0.05)*	0.80 (0.05)
Tnfrs11b	1.03 (0.11)	3.19 (0.27)*	3.11 (0.18)*	1.69 (0.06)*
Trib3	1.09 (0.11)	0.43 (0.06)*	0.76 (0.06)*	1.37 (0.07)
ICAM1	1.01 (0.07)	1.36 (0.03)*	1.35 (0.08)*	1.52 (0.16)*
VCAM1	1.00 (0.03)	0.59 (0.04)*	0.64 (0.04)*	0.70 (0.05)*
Wnt5a	1.01 (0.05)	2.31 (0.13)*	1.80 (0.09)*	0.79 (0.04)*
CXCL12	1.01 (0.08)	1.08 (0.04)	1.01 (0.04)	0.55 (0.01)*
IL1β	1.18 (0.11)	7.52 (0.52)*	5.99 (0.44)*	0.6 (0.06)*
IL8	1.25 (0.12)	7.95 (1.26)*	4.23 (0.38)*	1.24 (0.16)
PTGS2	1.01 (0.07)	3.92 (0.57)*	3.78 (0.39)*	1.49 (0.27)

The regulation of genes in precursors by DMSO, 25nM TCDD, 1µM PCB126 or 10µM PCB153 for 48h was assessed by qRT-PCR using the ΔΔCt method by reporting Ct of target gene of each treated cells to mean Ct of DMSO-treated cells and by using HPRT as reference gene for precursors. The fold induction is expressed as mean (SEM). For each gene, data were statistically compared by Kruskal Wallis test and if significant differences were found Mann-Whitney U test was applied between TCDD-, PCB126-, PCB153-treated cells vs DMSO cells *: $p<0.05$

Supplementary Material, Table 2: Gene regulation by pollutants in differentiated hMADS cells

Gene	DMSO	TCDD	PCB126	PCB153
AhR	1.02 (0.12)	0.77 (0.04)	0.73 (0.05)	0.91 (0.05)
CYP1B1	1.09 (0.12)	5.33 (0.29)*	6.44 (0.34)*	0.95 (0.12)
CYP19A1	1.01 (0.06)	0.86 (0.14)	0.87 (0.01)	0.61 (0.06)*
NPTX1	1.04 (0.05)	9.34 (1.63)*	11.23 (0.95)*	0.88 (0.05)
NQO1	1.00 (0.05)	1.14 (0.08)	1.39 (0.14)*	0.89 (0.04)
PAI2	1.06 (0.09)	5.96 (0.52)*	5.36 (0.24)*	0.92 (0.07)
FABP4	1.00 (0.05)	0.92 (0.03)	1.06 (0.13)	1.05 (0.08)
IGFBP3	1.09 (0.26)	1.28 (0.13)	1.50 (0.16)	0.99 (0.09)
STAT1	0.99 (0.09)	1.54 (0.15)*	1.61 (0.08)*	1.10 (0.04)
TGFβi	1.02 (0.12)	2.87 (0.51)*	3.36 (0.37)*	1.09 (0.07)
TSP1	1.03 (0.13)	1.51 (0.17)	1.66 (0.10)*	1.00 (0.08)
TNFRS11b	1.04 (0.17)	2.61 (0.32)*	2.97 (0.27)*	0.96 (0.06)
TRIB3	1.05 (0.21)	0.80 (0.09)	0.79 (0.11)	1.05 (0.18)
ICAM1	1.03 (0.09)	1.41 (0.21)	1.11 (0.06)	1.11 (0.08)
Vcam1	1.05 (0.18)	0.80 (0.09)	0.76 (0.10)	1.14 (0.10)
Wnt5a	1.03 (0.13)	1.56 (0.12)*	1.75 (0.14)*	1.06 (0.03)
CXCL12	1.07 (0.21)	1.34 (0.19)	1.25 (0.14)	1.09 (0.16)
IL1β	1.08 (0.09)	1.00 (0.08)	1.03 (0.07)	0.98 (0.08)
IL8	1.06 (0.09)	2.47 (0.23)*	2.65 (0.16)*	0.90 (0.08)
PTGS2	1.02 (0.09)	2.52 (0.26)*	2.27 (0.11)*	0.99 (0.02)

The regulation of genes in adipocytes by DMSO, 25nM TCDD, 1µM PCB126 or 10µM PCB153 for 48h was assessed by qRT-PCR using the ΔΔCt method by reporting Ct of target gene of each treated cells to mean Ct of DMSO-treated cells and by using TBP as reference gene for adipocytes. The fold induction is expressed as mean (SEM). For each gene, data were statistically compared by Kruskal Wallis test and if significant differences were found Mann-Whitney U test was applied between TCDD-, PCB126-, PCB153-treated cells vs DMSO-treated cells *: $p<0.05$

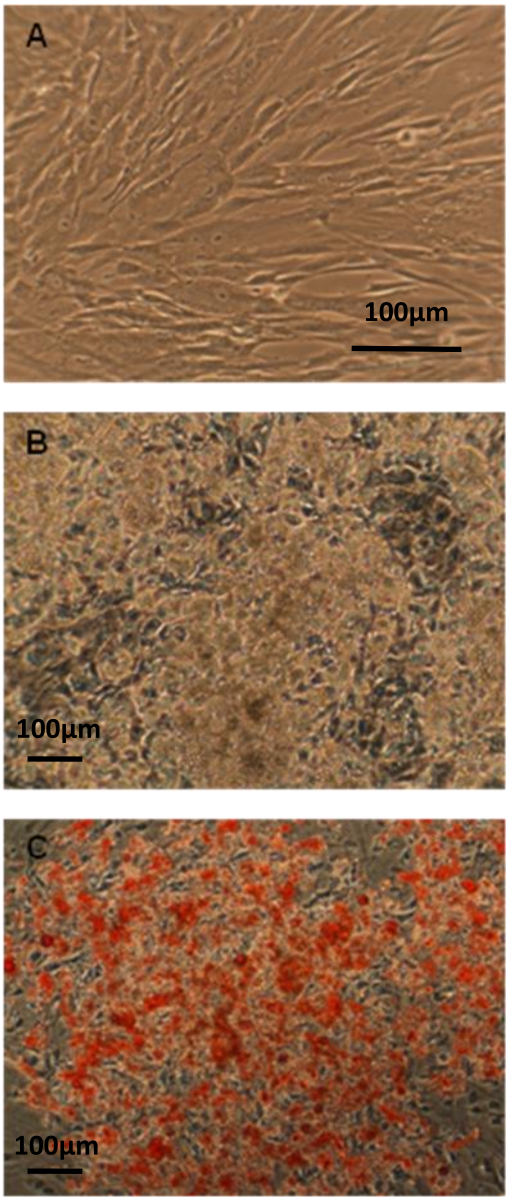

Supplementary Material, Figure 1: In vitro culture of hMADS cells

A: hMADS cells in proliferation medium; B: hMADS cells exhibited intracellular refringent vacuoles representing triglyceride droplets after 10 days in differentiation medium; C: the presence of triglycerides was assessed by Oil Red O staining
